# Cholecystectomy is associated with a higher risk of irritable bowel syndrome in the UK Biobank: a prospective cohort study

**DOI:** 10.3389/fphar.2023.1244563

**Published:** 2023-12-08

**Authors:** Jinyu Zhao, Liang Tian, Bin Xia, Ningning Mi, Qiangsheng He, Man Yang, Danni Wang, Siqing Wu, Zijun Li, Shiyong Zhang, Xianzhuo Zhang, Ping Yue, Yanyan Lin, Haitong Zhao, Baoping Zhang, Zelong Ma, Ningzu Jiang, Matu Li, Jinqiu Yuan, Peng Nie, Linzhi Lu, Wenbo Meng

**Affiliations:** ^1^ The First Clinical Medical School, Lanzhou University, Lanzhou, Gansu, China; ^2^ Department of General Surgery, The First Hospital of Lanzhou University, Lanzhou, Gansu, China; ^3^ Scientific Research Center, Big Data Center, The Seventh Affiliated Hospital, Sun Yat-Sen University, Shenzhen, Guangdong, China; ^4^ Clinical Research Center, The Seventh Affiliated Hospital, Sun Yat-Sen University, Shenzhen, Guangdong, China; ^5^ Guangdong Provincial Key Laboratory of Gastroenterology, Center for Digestive Disease, The Seventh Affiliated Hospital, Sun Yat-Sen University, Shenzhen, Guangdong, China; ^6^ School of Medicine, Shenzhen Campus of Sun Yat-Sen University, Shenzhen, China; ^7^ Evidence Based Social Science Research Center, School of Public Health, Lanzhou University, Lanzhou, China; ^8^ Department of Joint Surgery, The First Affiliated Hospital, Sun Yat-Sen University, Guangzhou, Guangdong, China; ^9^ Department of Gastroenterology, The First Hospital of Lanzhou University, Lanzhou, Gansu, China; ^10^ Department of Gastric Surgery, Gansu Wuwei Tumour Hospital, Wuwei Academy of Medical Science, Wuwei, Gansu, China; ^11^ Wuwei Oncology Hospital, Wuwei, Gansu, China

**Keywords:** cholecystectomy, irritable bowel syndrome, cohort study, prospective analysis, UK Biobank

## Abstract

**Background:** Recent studies have shown that bile acids are essential in irritable bowel syndrome (IBS) pathology, and cholecystectomy has direct effects on bile acid metabolism. However, whether cholecystectomy increases the risk of IBS remains unclear. We aimed to investigate the association between cholecystectomy and IBS risk in the UK Biobank (UKB).

**Methods:** This study is a prospective analysis of 413,472 participants who were free of IBS, inflammatory bowel disease, cancer, or common benign digestive tract diseases. We identified incidents of IBS through self-reporting or links to primary healthcare and hospitalization data. We evaluated hazard ratios (HRs) adjusted for sociodemographic characteristics, health behaviours, comorbidities, and medications.

**Results:** During a median follow-up period of 12.7 years, we observed 15,503 new cases of IBS. Participants with a history of cholecystectomy had a 46% higher risk of IBS than those without (HR = 1.46, 95% CI: 1.32–1.60), and further subtype analysis showed that the risk of IBS with diarrhoea was significantly higher than the risk of IBS without diarrhoea (HR = 1.71, 95% CI: 1.30–2.25 vs. HR = 1.42, 95% CI: 1.28–1.58). The overall covariate-adjusted HRs for IBS were similar between the group with both cholecystectomy and gallstones (HR = 1.45, 95% CI: 1.32–1.58) and the group with cholecystectomy without gallstones (HR = 1.50, 95% CI: 1.36–1.67) when the group without both cholecystectomy and gallstones was used as a reference. The overall covariate-adjusted HR was not significantly different in the group without cholecystectomy with gallstones (HR = 1.18, 95% CI: 0.95–1.47). The positive association of cholecystectomy with IBS risk did not change when stratifying the data based on age, sex, BMI, smoking, alcohol consumption, healthy diet, quality sleep, physical activity, type 2 diabetes, hypertension, hyperlipidaemia, mental illness, NSAID intake, or acid inhibitor intake. Sensitivity analyses, including propensity score matching analysis and lagging the exposure for two or four years, indicated that the effects were robust.

**Conclusion:** Cholecystectomy was associated with a higher risk of IBS, especially IBS with diarrhoea. Additional prospective randomized controlled and experimental studies are warranted to further validate the association and to explore the relevant biological mechanisms.

## 1 Introduction

Irritable bowel syndrome (IBS) is a chronic gastrointestinal dysfunction that is characterized by abdominal pain or discomfort accompanied by altered bowel habits without any other illness that would cause such symptoms. The Rome III and IV diagnostic criteria for functional gastrointestinal diseases divide IBS into four subtypes: IBS-constipation (IBS-C), IBS-diarrhoea (IBS-D), IBS-mixed (IBS-M), and IBS-unclassified (IBS-U) (Mearin et al., 2016). IBS is the most common gastrointestinal disorder (Chey et al., 2015), with a prevalence of up to 7%–21% in different regions of the world (Lovell and Ford, 2012). IBS significantly impacts patients’ quality of life and work efficiency while increasing the healthcare burden (Mikocka-Walus et al., 2008; Peery et al., 2015; Sperber et al., 2021; Peery et al., 2022). In the United States, IBS causes 3.1 million outpatient treatments and 5.9 million prescriptions annually, and the direct and indirect expenses of IBS exceed $20 billion (Koloski et al., 2001; Everhart and Ruhl, 2009; Agarwal and Spiegel, 2011). However, IBS treatments are limited, as the aetiology and biological mechanism have yet to be fully elucidated (Talley et al., 2015).

Previous epidemiological studies have found that 12%–43% of IBS-D patients exhibit excessive bile acid excretion, and nearly 25% of IBS-D patients suffer from bile acid malabsorption (Shin et al., 2013; Aziz et al., 2015; Camilleri, 2015; Peleman et al., 2017). Previous analysis of faecal bile acid profiles in IBS patients revealed that bile acid levels were higher in IBS-D patients and lower in IBS-C patients than in healthy controls (Shin et al., 2013; Camilleri et al., 2014). These findings imply that bile acids are essential in IBS pathology (Hou et al., 2022; Gu et al., 2023). Cholecystectomy has direct effects on bile acid metabolism (Yoon et al., 2019; Hu et al., 2022; Ma et al., 2022; Xu et al., 2023). Cross-sectional studies have shown a higher proportion of IBS in patients with cholelithiasis (de Jong et al., 2022) and in those with a history of cholecystectomy (Kennedy and Jones, 2000). McNally et al. (2008) found a positive association between biliary events (gallstones or cholecystectomy) and the risk of new IBS. However, whether cholecystectomy increases the risk of IBS is unknown.

In our opinion, it is essential to clarify the impact of cholecystectomy on the risk of IBS. If cholecystectomy is a risk factor for developing IBS, cholecystectomy should be chosen more carefully in clinical practice. In addition, this can verify the role of bile acids in the pathogenesis of IBS and guide further research on pathogenesis and treatment. Thus, we prospectively analyzed the association of cholecystectomy with the risk of IBS in a large-scale cohort including 413,472 participants.

## 2 Materials and methods

### 2.1 Study population and design

**FIGURE 1:**
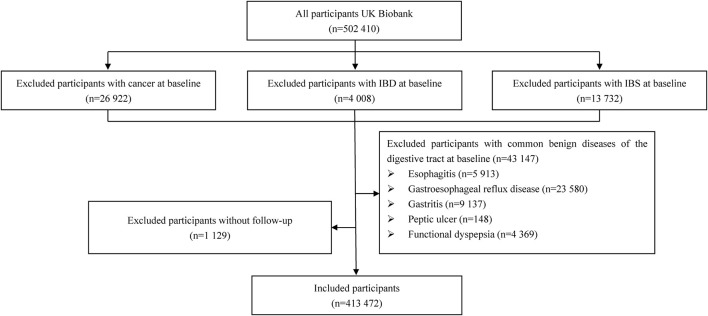
Flowchart of participant selection.

The study population included individuals in the UK Biobank (UKB), a large prospective cohort including over 500,000 participants aged between 37 and 73 years. The cohort was established from 2006 to 2010 at 22 assessment centres in England, Wales, and Scotland, and was regularly followed up to update the data. All participants completed a baseline questionnaire, including assessments of anthropometric measures, lifestyle, and medications. Additional information about the UKB has been published elsewhere (Sudlow et al., 2015). We excluded participants with a diagnosis of cancer, IBS, inflammatory bowel disease (IBD) at baseline, and those with missing follow-up data. Participants with the most common benign digestive system diseases with symptoms similar to IBS were also excluded, including patients with oesophagitis, gastroesophageal reflux disease (GERD), gastritis, peptic ulcer, or functional dyspepsia. The final analyses included 413,472 participants, as shown in Figure 1. All diagnoses were based on the International Classification of Disease-10 (ICD-10) diagnostic criteria (Supplementary Table S1).

### 2.2 Assessment of main exposures

The primary exposure was a history of cholecystectomy, including cholecystectomy for any reason based on self-reporting or linkage to primary healthcare and hospitalization records.

### 2.3 Assessment of main outcomes

The primary outcome was new onset IBS based on self-reporting or linkage to primary healthcare and hospitalization data. New onset IBS within our study was diagnosed using the Rome III criteria (Longstreth et al., 2006), which are based on reports of symptoms such as recurrent abdominal pain or discomfort at least 3 days per month in the last 3 months associated with two or more of the following: 1) symptoms improved after defecation; 2) onset associated with a change in the frequency of stool; and 3) onset associated with a change in the form of stool. Moreover, the criteria had to be met for the last 3 months, with symptom onset occurring at least 6 months prior to diagnosis. The diagnosis of IBS was determined using the ICD-10 codes (K58, Supplementary Table S1). The secondary outcome was the subtype of IBS, which was classified according to the online questionnaire for IBS patients in the UKB (Fild ID: 21074). Based on whether patients had diarrhoea symptoms at the initial diagnosis, IBS was classified into two subtypes: IBS with diarrhoea and IBS without diarrhoea. When asked, “When your IBS symptoms first began, did you have diarrhea symptoms?” Patients who answered “Yes” were classified as IBS with diarrhoea, and patients who answered “No” or “Prefer not to answer” were classified as IBS without diarrhoea.

### 2.4 Assessment of covariates

Based on previous epidemiological studies, sociodemographic characteristics, lifestyle factors, comorbidities, and medications were considered to be covariates. The sociodemographic factors included age (continuous variable), sex (female or male), race (white or nonwhite), education level (college/university or noncollege/university), Townsend Deprivation Index (continuous variable), and body mass index (BMI) (continuous variable). The Townsend Deprivation Index was used to evaluate socioeconomic status. The lifestyle factors included physical activity (continuous variable), smoking (yes or no), alcohol consumption (yes or no), healthy diet (yes or no), and quality sleep (yes or no). Physical activity was evaluated using metabolic equivalent (MET). The comorbidities included type 2 diabetes (yes or no), hypertension (yes or no), hyperlipidaemia (yes or no), mental illness (yes or no), and gallstones (yes or no). The presence of gallstones was determined using the ICD-10 codes for cholelithiasis (K80, Supplementary Table S1). The medications included nonsteroidal anti-inflammatory drug (NSAID) intake (yes or no), acid inhibitor intake (yes or no), hypoglycaemic drug intake (yes or no), hypolipidaemic drug intake (yes or no), antihypertensive drug intake (yes or no), vitamin supplement intake (yes or no), and mineral supplement intake (yes or no). The definitions of a healthy diet, quality sleep, mental illness, and medications (e.g., NSAIDs, acid inhibitors, and hypolipidaemic drugs) were based on previous studies (Watson et al., 2015; Lourida et al., 2019; Liu et al., 2022; Nishiyama and Fujikawa, 2023). The details are described in Supplementary Table S2.

### 2.5 Statistical analysis

For the baseline characteristics of the study population, normally distributed continuous variables are presented as the means and standard deviations (SDs), nonnormally distributed continuous variables are presented as medians and interquartile ranges (IQRs), and categorical variables are presented as numbers and percentages. The independent samples Student’s *t*-test was used to compare normally distributed continuous variables, and the Mann-Whitney U test was used to compare nonnormally distributed continuous variables. The frequencies of categorical variables were compared using the Pearson χ^2^ test.

The incidence rate (IR) of IBS was calculated as the number of events per 10,000 person-years, and hazard ratios (HRs) with 95% confidence intervals (CIs) were calculated using the multivariable Cox regression model. The models were adjusted for covariates as follows: 1) age and gender were adjusted in model 1; 2) race, education level, Townsend Deprivation Index, BMI, physical activity, smoking, alcohol drinking, healthy diet, and sleep quality were additionally adjusted in model 2; and 3) type 2 diabetes, hypertension, hyperlipidaemia, mental illness, gallstones, intake of NSAIDs, acid inhibitors, hypoglycaemic drugs, hypolipidaemic drugs, antihypertensive drugs, vitamin supplements, and mineral supplements were further adjusted in model 3. To facilitate a clinically understandable presentation of the associations, we calculated the number needed to harm (NNH) and risk difference (RD) using a previously described method (Altman and Andersen, 1999).

We evaluated the potential moderating effects of sex, age, BMI, smoking, alcohol consumption, healthy diet, quality of sleep, physical activity, type 2 diabetes, hypertension, hyperlipidaemia, mental illness, intake of NSAIDs, and acid inhibitors using a stratified analysis and interaction testing with a likelihood ratio test in subgroup analyses. We also performed sensitivity analyses to assess the robustness of the results, including propensity score matching analysis and lagging exposure for two or four years.

For the hypothesis test, a two-tailed *p*-value < 0.05 was considered statistically significant. All analyses were performed using R version 4.2.2.

## 3 Results

### 3.1 Baseline characteristics

This study included 413,472 participants, 13,179 of whom had a history of cholecystectomy. During the median follow-up period of 12.7 years, we observed 15,503 new cases of IBS. Table 1 describes the baseline characteristics of the study participants. Individuals who had undergone cholecystectomy were more likely to be female, White, smokers, and older adults and had a lower level of education, less physical activity, and poor-quality sleep than those without cholecystectomy. Additionally, individuals with cholecystectomy had a higher BMI and a higher proportion of comorbidities, including type 2 diabetes, hypertension, hyperlipidaemia, and mental illness. Additionally, these individuals had higher use of medications, such as NSAIDs, acid inhibitors, hypoglycaemic drugs, hypolipidaemic drugs, antihypertensive drugs, and vitamin supplements.

**TABLE 1:** Baseline characteristics according to cholecystectomy in UK Biobank cohort.

Characteristic	Total	Cholecystectomy		*p*-value
	(N = 413472)	No (N = 400293)	Yes (N = 13179)	
Mean (SD) age (years)	56.09 (8.13)	55.99 (8.14)	59.29 (7.21)	<0.001*
Male, No. (%)	192,460 (46.5)	189,448 (47.3)	3,012 (22.9)	<0.001
White race, No. (%)	388,091 (93.9)	375,372 (93.8)	12,719 (96.5)	<0.001
College/University, No. (%)	137,760 (33.3)	134,417 (33.6)	3,343 (25.4)	<0.001
Median (IQR) Townsend Deprivation Index	12.91 (15.41)	12.91 (15.40)	13.14 (15.53)	0.010^※^
Smoking, No. (%)	182,261 (44.1)	176,197 (44.0)	6,064 (46.0)	<0.001
Alcohol drinking, No. (%)	335,429 (81.1)	326,055 (81.5)	9,374 (71.1)	<0.001
Healthy diet, No. (%)	264,817 (64.0)	255,963 (63.9)	8,854 (67.2)	<0.001
Quality sleep, No. (%)	174,907 (42.3)	170,096 (42.5)	4,811 (36.5)	<0.001
Median (IQR) Physical activity (MET hours/week)	41.00 (32.87)	41.00 (33.03)	39.75 (28.91)	<0.001^※^
Mean (SD) BMI	27.32 (4.74)	27.25 (4.69)	29.68 (5.60)	<0.001*
Type 2 diabetes, No. (%)	69,965 (16.9)	66,730 (16.7)	3,235 (24.5)	<0.001
Hypertension, No. (%)	299,461 (72.4)	289,135 (72.2)	10,326 (78.4)	<0.001
Hyperlipidemia, No. (%)	186,870 (45.2)	179,529 (44.8)	7,341 (55.7)	<0.001
Mental illness, No. (%)	26,140 (6.3)	25,119 (6.3)	1,021 (7.7)	<0.001
gallstones, No. (%)	9,365 (2.3)	1,534 (0.4)	7,831 (59.4)	<0.001
NASID intake, No. (%)	164,738 (39.8)	158,220 (39.5)	6,518 (49.5)	<0.001
Acid inhibitor intake, No. (%)	24,536 (5.9)	22,745 (5.7)	1,791 (13.6)	<0.001
Hypoglycemic drug intake, No. (%)	5,406 (1.3)	5,092 (1.3)	314 (2.4)	<0.001
Hypolipidemic drug intake, No. (%)	69,353 (16.8)	66,348 (16.6)	3,005 (22.8)	<0.001
Antihypertensive drug intake, No. (%)	80,371 (19.4)	76,542 (19.1)	3,829 (29.1)	<0.001
Vitamin supplement intake, No. (%)	60,203 (14.6)	58,183 (14.5)	2,020 (15.3)	0.012
Mineral supplement intake, No. (%)	87,765 (21.2)	84,893 (21.2)	2,872 (21.8)	0.109

Note: Normally distributed continuous variables displayed as the means (SDs), nonnormally distributed continuous variables displayed as medians (IQRs), and categorical variables displayed as numbers (percentages). *: *p*-value calculated using independent samples Student’s *t*-test. ※: *p*-value calculated using Mann-Whitney U test. The remaining *p*-value calculated using Pearson χ^2^ test. Abbreviations: SD, standard deviation; IQR, interquartile range; MET, metabolic equivalent; BMI, body mass index; NASID, nonsteroidal anti-inflammatory drug.

### 3.2 Cholecystectomy and the risk of incident IBS

Table 2 shows the association between cholecystectomy and the risk of IBS and its subtype. We identified 883 cases of IBS in the cholecystectomy group, including 113 cases with diarrhoea and 770 cases without diarrhoea; additionally, we identified 14,620 cases of IBS in the noncholecystectomy group, including 1,639 cases with diarrhoea and 12,981 cases without diarrhoea. In the Cox regression model adjusting for age and sex, participants who underwent cholecystectomy had a 59% higher risk of all IBS than those without cholecystectomy (Table 2, HR = 1.59, 95% CI: 1.49–1.70). This association was slightly attenuated after adjusting for socioeconomic characteristics and lifestyle factors (Table 2, HR = 1.56, 95% CI: 1.45–1.67). After further adjusting the model for comorbidities and medication factors, the HR slightly decreased (Table 2, HR = 1.46, 95% CI: 1.32–1.60). For ease of interpretation, we calculated the NNH and RD based on the HR of adjusted model 3 and the IR of all IBS in individuals who did not undergo cholecystectomy (Supplementary Figure S1). For every 365 (95% CI: 350–402), 79 (95% CI: 74–92), and 42 (95% CI: 39–50) individuals who underwent cholecystectomy, one case of IBS would occur over 1, 5, and 10 years, respectively (Supplementary Figure S1). The multivariable Cox regression analysis between cholecystectomy and IBS subtype revealed that cholecystectomy significantly increased the risk of IBS with diarrhoea and IBS without diarrhoea by 71% (Table 2, HR = 1.71, 95% CI: 1.30–2.25) and 42% (Table 2, HR = 1.42, 95% CI: 1.28–1.58), respectively.

**TABLE 2:** Risk of IBS according to cholecystectomy.

Group	Cholecystectomy		*p*-value
	No (N = 400293)	Yes (N = 13179)	
All IBS			
Case/person-years	14,620/4964909	883/162107	
IR (per 10,000 person-years)	29.45	54.47	
Multivariate-adjusted HR (95% CI)			
Adjusted model 1^a^	1.00 (Reference)	1.59 (1.49–1.70)	<0.001
Adjusted model 2^b^	1.00 (Reference)	1.56 (1.45–1.67)	<0.001
Adjusted model 3^c^	1.00 (Reference)	1.46 (1.32–1.60)	<0.001
IBS with diarrhea			
Case/person-years	1,639/4964909	113/162107	
IR (per 10,000 person-years)	3.30	6.97	
Multivariate-adjusted HR (95% CI)			
Adjusted model 1^a^	1.00 (Reference)	1.87 (1.54–2.26)	<0.001
Adjusted model 2^b^	1.00 (Reference)	1.79 (1.48–2.18)	<0.001
Adjusted model 3^c^	1.00 (Reference)	1.71 (1.30–2.25)	<0.001
IBS without diarrhea			
Case/person-years	12,981/4964909	770/162107	
IR (per 10,000 person-years)	26.15	47.50	
Multivariate-adjusted HR (95% CI)			
Adjusted model 1^a^	1.00 (Reference)	1.56 (1.45–1.68)	<0.001
Adjusted model 2^b^	1.00 (Reference)	1.53 (1.42–1.64)	<0.001
Adjusted model 3^c^	1.00 (Reference)	1.42 (1.28–1.58)	<0.001

^a^ Age (continuous variable) and gender (female or male) were adjusted in model 1.

^b^ Race (white or nonwhite), education level (college/university or non-college/university), Townsend Deprivation Index (continuous variable), physical activity (continuous variable), smoking (yes or no), alcohol drinking (yes or no), healthy diet (yes or no), quality sleep (yes or no), and BMI (continuous variable) were additionally adjusted in model 2.

^c^ Type 2 diabetes (yes or no), hypertension (yes or no), hyperlipidemia (yes or no), mental illness (yes or no), gallstones (yes or no), NASID intake (yes or no), acid inhibitor intake (yes or no), hypoglycemic drug intake (yes or no), hypolipidemic drug intake (yes or no), antihypertensive drug intake (yes or no), vitamin supplement intake (yes or no), and mineral supplement intake (yes or no) were further adjusted in model 3. Abbreviations: IBS, irritable bowel syndrome; IR, incidence rate; HR, hazard ratio; CI, confidence interval.

To further investigate the relative or combined effects of cholecystectomy and gallstones on the development of IBS, we categorized participants into the following four groups according to the status of cholecystectomy and presence of gallstones: noncholecystectomy without gallstones, noncholecystectomy with gallstones, cholecystectomy without gallstones, and cholecystectomy with gallstones. Then, we assessed the risk of incident IBS among the four groups (Table 3). Compared with the reference group (noncholecystectomy without gallstones), we observed significantly higher HRs of IBS in the noncholecystectomy group with gallstones, cholecystectomy group without gallstones, and cholecystectomy group with gallstones in adjusted models 1 and 2 (Table 3). Interestingly, the HRs were higher and similar in the cholecystectomy group with or without gallstones. In contrast, the HR was lower in the noncholecystectomy group with gallstones. After adjusting for all the covariates, the HR of IBS was 1.18 for the noncholecystectomy group with gallstones (*p* = 0.127), 1.50 for the cholecystectomy group without gallstones (*p* < 0.001), and 1.45 for the cholecystectomy group with gallstones (*p* < 0.001) when using the noncholecystectomy group without gallstones as the reference group (Table 3, adjusted model 3).

**TABLE 3:** Risk of incident IBS according to the status of cholecystectomy and gallstones.

Group	Non-cholecystectomy			Cholecystectomy			
	Without gallstones (N = 398759)	With gallstones (N = 1,534)	*p*-value	Without gallstones (N = 5,348)	*p*-value	With gallstones (N = 7,831)	*p*-value
Case/person-years	14,538/4946298	82/18611		377/65956		506/96152	
Multivariate-adjusted HR (95% CI)							
Adjusted model 1^a^	1.00 (Reference)	1.35 (1.09–1.68)	0.007	1.63 (1.47–1.80)	<0.001	1.57 (1.44–1.72)	<0.001
Adjusted model 2^b^	1.00 (Reference)	1.32 (1.06–1.64)	0.013	1.60 (1.44–1.77)	<0.001	1.53 (1.40–1.68)	<0.001
Adjusted model 3^c^	1.00 (Reference)	1.18 (0.95–1.47)	0.127	1.50 (1.36–1.67)	<0.001	1.45 (1.32–1.58)	<0.001

^a^ Age (continuous variable) and gender (female or male) were adjusted in model 1.

^b^ Race (white or nonwhite), education level (college/university or non-college/university), Townsend Deprivation Index (continuous variable), physical activity (continuous variable), smoking (yes or no), alcohol drinking (yes or no), healthy diet (yes or no), quality sleep (yes or no), and BMI (continuous variable) were additionally adjusted in model 2.

^c^ Type 2 diabetes (yes or no), hypertension (yes or no), hyperlipidemia (yes or no), mental illness (yes or no), NASID intake (yes or no), acid inhibitor intake (yes or no), hypoglycemic drug intake (yes or no), hypolipidemic drug intake (yes or no), antihypertensive drug intake (yes or no), vitamin supplement intake (yes or no), and mineral supplement intake (yes or no) were further adjusted in model 3. Abbreviations: IBS, irritable bowel syndrome; IR, incidence rate; HR, hazard ratio; CI, confidence interval.

### 3.3 Subgroup analysis

**FIGURE 2:**
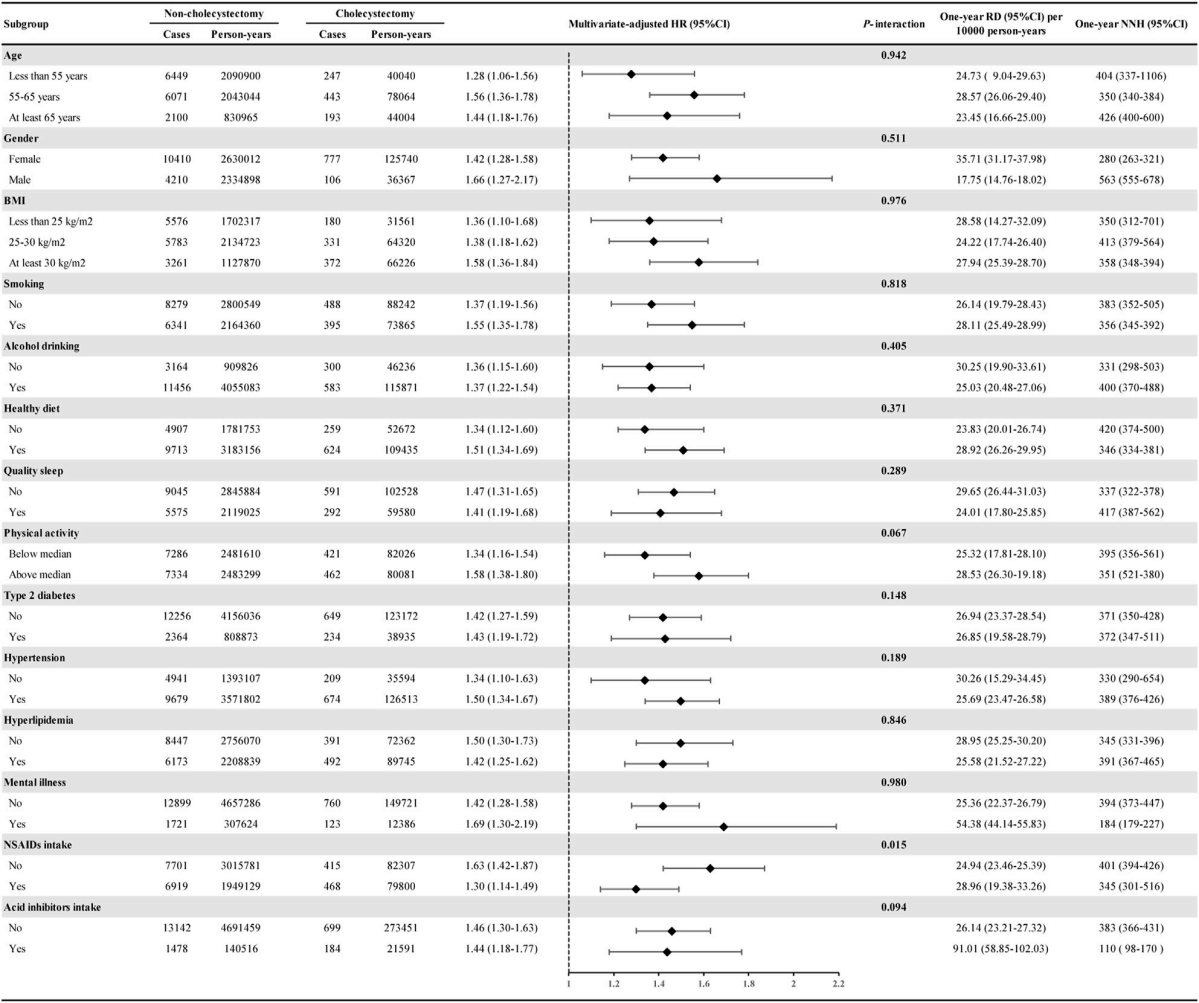
Subgroup analyses of cholecystectomy and the risk of IBS. Estimated effects were based on the adjusted Cox proportional hazards regression model adjusting for age (continuous variable), gender (female or male), race (White or non-White), education level (college/university or non-college/university), Townsend Deprivation Index (continuous variable), physical activity (continuous variable), smoking (yes or no), alcohol drinking (yes or no), healthy diet (yes or no), quality sleep (yes or no), BMI (continuous variable), type 2 diabetes (yes or no), hypertension (yes or no), hyperlipidemia (yes or no), mental illness (yes or no), gallstones (yes or no), NASID intake (yes or no), acid inhibitor intake (yes or no), hypoglycemic drug intake (yes or no), hypolipidemic drug intake (yes or no), antihypertensive drug intake (yes or no), vitamin supplement intake (yes or no), and mineral supplement intake (yes or no). The HRs and 95% CIs for incident IBS in the cholecystectomy group were compared with the non-cholecystectomy group (as a reference). Abbreviations: IBS, irritable bowel syndrome. RD, risk difference. NNH, number needed to harm. HR, hazard ratio. CI, confidence interval.

In subgroup analyses, cholecystectomy was associated with higher IBS risk in all subgroups, and the multivariate-adjusted HRs ranged from 1.28 to 1.69 (Figure 2). The risk of IBS did not differ based on age, gender, BMI, smoking, alcohol consumption, healthy diet, quality sleep, physical activity, type 2 diabetes, hypertension, hyperlipidaemia, mental illness, or acid inhibitor intake, but did differ based on the regular use of NSAIDs (*P*-interaction = 0.015). Specifically, cholecystectomy was associated with a 63% higher risk of IBS in participants without regular use of NSAIDs (HR = 1.63, 95% CI: 1.42–1.87) and a 30% higher risk in participants with regular use of NSAIDs (HR = 1.30, 95% CI: 1.14–1.49).

### 3.4 Sensitivity analysis

The main results were found to be robust across various sensitivity analyses (Supplementary Table S3). We generated a cohort containing 29,811 participants through propensity score matching of all covariates. Cholecystectomy increased the risk of IBS by 42% in this cohort (HR = 1.42, 95% CI: 1.28–1.59). In addition, we did not find significant changes in the cholecystectomy and IBS risk after lagging the exposure for 2 years (HR = 1.45, 95% CI: 1.32–1.60) or for 4 years (HR = 1.43, 95% CI: 1.30–1.59).

## 4 Discussion

We conducted a prospective cohort study involving more than 400,000 participants to examine the association between cholecystectomy and the risk of IBS. We found that cholecystectomy was associated with a 46% higher risk of IBS. The risks of IBS between cholecystectomy with gallstones and without gallstones were similar. This finding means that patients after cholecystectomy should be more alert to IBS.

A series of studies have observed the relationships among gallstones, cholecystectomy, and IBS. Many studies have reported that IBS was highly prevalent in patients with gallstones and cholecystectomy (Kennedy and Jones, 2000; de Jong et al., 2022), but these cross-sectional data did not prove a causal relationship. Additionally, IBS increased the incidence rate of unnecessary cholecystectomies (Corazziari et al., 2008) and was associated with poor outcomes after cholecystectomy (Kirk et al., 2011; de Jong et al., 2022). Notably, Corazziari et al. (2008) observed that IBS patients were more aware of gallstones than healthy controls and that the prevalence of gallstones in IBS patients unaware of their gallbladder status was not significantly different from that in controls. This finding supported that IBS may increase the risk of cholecystectomy mainly due to abdominal pain symptoms, awareness of gallstones, and inappropriate surgical indications, instead of increasing the risk of stone formation. However, it is unclear whether cholecystectomy increases the risk of IBS. Heaton et al. (1993) reported changes in defecation habits after cholecystectomy in women, but it was not identified as IBS. To our knowledge, only one previous study prospectively analyzed the effect of cholecystectomy on the risk of developing IBS. McNally et al. (2008) followed 1908 participants in the Minnesota cohort and found that biliary events (defined as gallstones or a history of cholecystectomy) were associated with a higher risk of new IBS. In detail, the incidence rate of new IBS was 16% in participants with biliary events and only 8.5% in those without biliary events. However, this study did not differentiate between gallstones and cholecystectomy in the analysis and was limited in sample size. Therefore, it is necessary to assess the specific contribution of cholecystectomy itself (but not gallstones) to the risk of IBS using large-scale cohort data. To our knowledge, this is the first and largest-scale cohort study to prospectively analyze the association between cholecystectomy and the risk of IBS in the general population.

We observed a robust and prevalent positive correlation between cholecystectomy and the risk of IBS in the present study. In multivariate Cox regression analysis, cholecystectomy significantly increased the risk of IBS. Even after controlling for many potential confounding factors, including demographic and sociological characteristics, healthy lifestyle factors, comorbid diseases, and medication status, the risk of IBS was still significantly increased. Furthermore, different subgroup analyses and multiple sensitivity analyses consistently showed that cholecystectomy was associated with a higher risk of IBS. Notably, herein, patients with a history of cholecystectomy who reported regular use of NSAIDs at baseline had a lower risk of IBS than patients without regular use of NSAIDs. This finding seems to be inconsistent with previous reports. Keszthelyi et al. (2012) investigated medications in the 6 months preceding the onset of IBS symptoms, including proton pump inhibitors, NSAIDs, selective serotonin reuptake inhibitors, diuretics, and angiotensin-converting enzyme inhibitors, and found that NSAIDs were more frequently used in IBS patients than in controls. Recurrent and treatment-refractory abdominal pain and the extragastrointestinal pain-related symptoms commonly observed in IBS may explain the increased use of NSAIDs (Talley et al., 1995). We hypothesize that NSAIDs may reduce the cholecystectomy rate and delay IBS diagnosis by relieving abdominal and extragastrointestinal pain in the present study. Although NSAIDs are known to alter intestinal physiology, especially barrier function (Kerckhoffs et al., 2010), the causal relationship between NSAIDs and developing IBS is unclear. The associations and mechanisms among NSAIDs, cholecystectomy, and the risk of IBS need further research to clarify. Additionally, we investigated the relative or combined effects of cholecystectomy and gallstones on developing IBS. We found that compared with the noncholecystectomy and without gallstones group, cholecystectomy significantly increased the risk of IBS regardless of whether the participants had gallstones. Interestingly, we observed that gallstones alone without cholecystectomy did not seem to increase the risk of IBS. However, this finding needs to be further verified, as cholecystectomy was associated with a higher risk of IBS in adjusted models 1 and 2, and the number of individuals without cholecystectomy but with gallstones was limited. In all, our study supported that cholecystectomy may independently contribute to developing IBS.

The mechanism underlying the relationship between cholecystectomy and IBS is unclear. Changes in intestinal function and abdominal pain related to intestinal function are characteristic symptoms of IBS (Mearin et al., 2016). Previous studies have revealed the role of gut microbiota dysbiosis, intestinal mucosal inflammation, and bile salt metabolism disorder in the pathogenesis of IBS (Inagaki et al., 2006; Holtmann et al., 2016; Li et al., 2017). Rajilić-Stojanović et al. (2015) reported that the ratio of *Firmicutes* to *Bacteroidetes* widely increased in IBS patients, along with a higher level of *Clostridium XIVa*. This *Clostridium* was correlated with the release of enteric serotonin (5-HT), which is a crucial neuroendocrine factor in IBS pathogenesis (Golubeva et al., 2017; Fung et al., 2019). It is worth noting that alterations in intestinal microbiota and abnormal bile acid metabolism have been confirmed after cholecystectomy. The gallbladder is where bile acids are stored, concentrated, and released rhythmically (Sylwestrowicz and Shaffer, 1988). After cholecystectomy, uncondensed bile acids flow continuously into the intestine and are propelled into the colon by the migrating motor complex (MMC), which results in a higher-than-normal concentration (Ford et al., 1992). Excessive bile acid increases mucosal permeability and inflammation, affects the secretory function of the colon, and stimulates high-amplitude colonic contraction, which accelerates colonic transport and easily leads to diarrhea. Previous studies have found that almost 12%–43% of IBS-D patients show excessive excretion of bile acids, and approximately 25% of IBS-D patients suffer from bile acid malabsorption (Shin et al., 2013; Aziz et al., 2015; Camilleri, 2015; Peleman et al., 2017). Excessive bile acids in intestinal feces were considered to be a critical pathogenic factor of IBS-D (Shin et al., 2013; Bajor et al., 2015; Camilleri et al., 2015; Ford et al., 2020), which might explain why IBS with diarrhea was more common than without diarrhoea after cholecystectomy in the present study. Interestingly, two recent studies have reported the mechanism of postcholecystectomy diarrhoea (PCD). Xu et al. (2022) performed 16sRNA sequencing of the gut microbiota in PCD patients and found that the bacterial composition was remarkably shifted in PCD patients, mainly in the dominant phyla *Firmicutes* and *Bacteroidetes*. Xu et al. (2023) further established a humanized gut microbiome mouse model by faecal microbiota transplantation to explore the diarrhoea inducible effects of gut microbiota and observed that altered gut microbiota after cholecystectomy contributes to PCD by promoting secondary bile acids in the colon, which stimulates colonic 5-HT and increases colon motility. The genuine relationship between IBS and PCD is highly anticipated. Collectively, the disturbance of the bile acid-gut microbiota axis is likely a vital pathogenesis pathway of cholecystectomy-induced IBS, especially in the subtype with diarrhoea (Shin et al., 2013; Gahan and Hill, 2014; Bajor et al., 2015; Camilleri et al., 2015; Holtmann et al., 2016; Ford et al., 2020; Camilleri, 2021). However, direct evidence of the mechanisms is limited, and further research is necessary.

There were some strengths in the present prospective study. First, we reported for the first time the independent effect of cholecystectomy on IBS risk in a large-scale and long-term follow-up cohort. We observed that cholecystectomy was associated with a higher risk of IBS after controlling for several potential confounding factors related to cholecystectomy and IBS. Second, we investigated the relative or combined effects of cholecystectomy and gallstones on developing IBS and confirmed the independent contribution of cholecystectomy on IBS risk. Third, a series of subgroup analyses and sensitivity analyses were conducted to verify the universality and robustness of the results. Nevertheless, several limitations should be discussed. First, as an observational study, we cannot exclude the residual confounding effect. However, the influence would be minor, as we have comprehensively adjusted for major risk factors of IBS. Second, we did not have information on the date of cholecystectomy, so we could not assess the short-term and long-term IBS risk after cholecystectomy. Third, misclassification of exposure during follow-up might exist because cholecystectomy was only evaluated once at baseline. However, misclassification would underestimate the true effects as there were some participants with cholecystectomy (with a high risk of IBS) in the control group. Last, this study was conducted in the UK Biobank cohort, which is a cohort of European descent. Therefore, our results may not be generalizable to populations with distinct ancestry. Future studies are needed that test these relations in more diverse populations.

## 5 Conclusion

We found that cholecystectomy was an independent risk factor for the development of IBS in this large-scale and long-term follow-up cohort study. Cholecystectomy significantly increased the risk of IBS, especially in the subtype of IBS with diarrhoea. The positive association between cholecystectomy and the risk of IBS was widespread, and it seemed to be unaffected by age, sex, metabolic status (e.g., BMI, type 2 diabetes, hypertension, and hyperlipidaemia), lifestyle factors (e.g., diet, smoking status, alcohol consumption, physical activity, and sleep), and medications (e.g., acid inhibitors and NSAIDs). We also observed that compared to the control group (noncholecystectomy and without gallstones), gallstones alone without cholecystectomy seemed to only slightly increase or even not increase the risk of IBS, while cholecystectomy with or without gallstones significantly increased the risk of IBS. These findings suggested that cholecystectomy may independently contribute to the development of IBS. Therefore, cholecystectomy should be selected after careful consideration of surgical indications, since this surgery would act as a predisposing factor for IBS. In addition, the relationship between cholecystectomy and IBS needs to be further verified in prospective randomized controlled and experimental studies. Moreover, it is of great clinical significance to explore the risk assessment methods and biological mechanisms of cholecystectomy-related IBS.

## Data Availability

The data analyzed in this study is subject to the following licenses/restrictions: The data used in this study are available from UK Biobank, but access is restricted due to licensing agreements. Interested parties can apply for access through the UK Biobank website (https://www.ukbiobank.ac.uk/). The authors are willing to make the data available upon reasonable request and with permission from UK Biobank. Requests to access these datasets should be directed to https://www.ukbiobank.ac.uk/.

## Abbreviations

IBS, irritable bowel syndrome; UKB, UK Biobank; IR, incidence rate; RD, rate difference; NNH, number needed to harm; HR, hazard ratio; CI, confidence interval; SD, standard deviation; IQR, interquartile range; IBD, inflammatory bowel disease; GERD, gastroesophageal reflux disease; ICD-10, International Classification of Disease-10; MET, metabolic equivalent; BMI, body mass index; NASID, nonsteroidal anti-inflammatory drug; MMC, migrating motor complex; PCD, post-cholecystectomy diarrhea.
