# Supplementary material for: Cholecystectomy is associated with a higher risk of irritable bowel syndrome in the UK Biobank: a prospective cohort study
Source: Front Pharmacol. 2023 Dec 8;14:1244563. doi: 10.3389/fphar.2023.1244563 (PMC10749201; doi:10.3389/fphar.2023.1244563)
Supplement: Supplementary file 1 [file DataSheet1.docx]

Supplementary Material

Cholecystectomy is associated with a higher risk of irritable bowel syndrome in the UK Biobank: a prospective cohort study

**Jinyu Zhao1,2†, Liang Tian1,2†, Bin Xia3,4,5, Ningning Mi1,2, Qiangsheng He3,4,5, Man Yang5, Danni Wang3, Siqing Wu6, Zijun Li7, Shiyong Zhang8, Xianzhuo Zhang1,2, Ping Yue2, Yanyan Lin2, Haitong Zhao7, Baoping Zhang1,2, Zelong Ma1,2, Ningzu Jiang1,2, Matu Li1,9, Jinqiu Yuan3,4,5, Peng Nie10*, Linzhi Lu11*, Wenbo Meng1,2***

*** Correspondence:**

Wenbo Meng; E-mail: mengwb@lzu.edu.cn

Peng Nie; E-mail: nie.peng2008@163.com

Linzhi Lu; E-mail:lulinzh12006@163.com

# Supplementary Data

None.

# Supplementary Figures and Tables

##
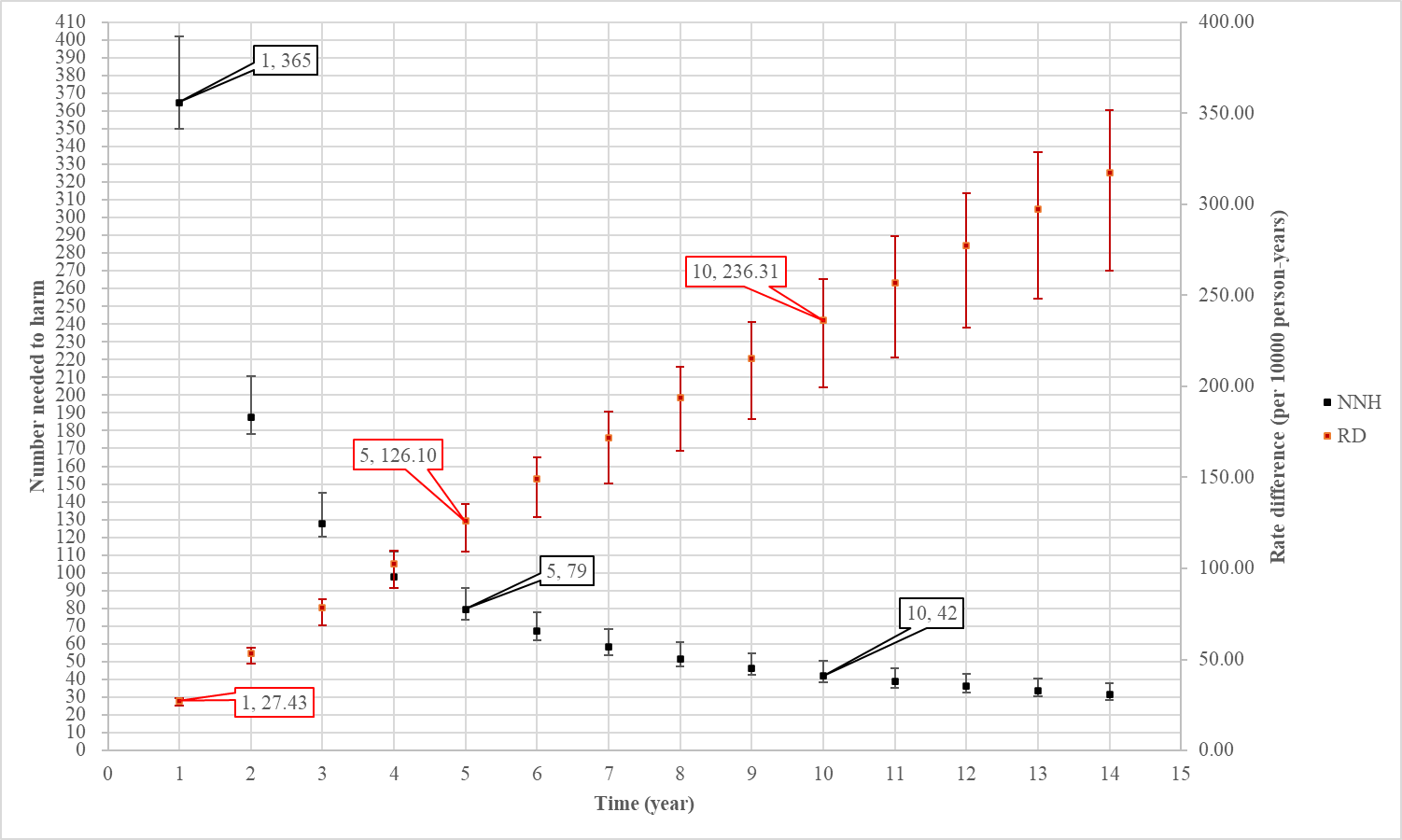
Supplementary Figures

**Supplementary Figure S1.** The NNHs and RDs of IBS between cholecystectomy and non-cholecystectomy during follow-up years. The estimated number needed to harm was based on the fully adjusted HR of cholecystectomy versus non-cholecystectomy (1.46, 95% CI: 1.32-1.60) and IBS rate in the non-cholecystectomy group (29.45 cases/10 000 person-years), with the method described by Altman et al^1^. Abbreviations: IBS, irritable bowel syndrome. RD, risk difference. NNH, number needed to harm.

## Supplementary Tables

**Supplementary Table S1.** ICD-10 codes and Field ID defining diseases in UKB.

| **Disease** | **ICD-10** | **Data field ID in UKB** |
| --- | --- | --- |
| Irritable bowel syndrome | K58 | 131638, 131639 |
| Cholelithiasis | K80 | 131674, 131675 |
| Crohn’s disease | K50 | 131626, 131627 |
| Ulcerative colitis | K51 | 131628, 131629 |
| esophagitis | K20 | 131582, 131583 |
| Gastro-esophageal reflux disease | K21 | 131584, 131585 |
| Gastric ulcer | K25 | 131590, 131591 |
| Duodenal ulcer | K26 | 131592, 131593 |
| Peptic ulcer, site unspecified | K27 | 131594, 131595 |
| Gastritis and duodenitis | K29 | 131596, 131597 |
| Dyspepsia | K30 | 131600, 131601 |

Abbreviations: ICD, international classification disease. UKB, UK Biobank.

**Supplementary Table S2.** Covariates about healthy diet, quality sleep, metabolic syndrome, mental illness, and medication status definitions and field ID in UKB.

| **Covariate** | **Source and definition** | **Data field ID** |
| --- | --- | --- |
| A healthy diet^2-4^ | UK Biobank Food Frequency Questionnaire at baseline;  At least 4 of the following 8 food groups:  1. Vegetables: ≥ 3 servings/day  2. Fruits: ≥ 3 servings/day  3. Fish: ≥2 servings/week  4. Poultry: ≥2 servings/week  5. Processed meats: ≤ 1 serving/week  6. Unprocessed red meats: ≤ 1.5 servings/week  7. Tea: 1-6 cups/day  8. Coffee: 1-4 cups/day | 1289, 1299, 1309,  1319, 1329, 1339, 1349, 1359, 1369, 1379, 1389, 1488, 1498 |
| Quality sleep^5^ | UK Biobank touchscreen questionnaire at baseline;  Without any one of the following 4 tips:  1. Total sleep time: >9 or <7 hours/day  2. Insomnia or sleeplessness usually  3. Snoring  4. Nap during day all the time or often | 1160, 1190, 1200,  1210 |
| Mental illness | UK Biobank touchscreen questionnaire at baseline;  With any one of the followings:  1. Bipolar disorder  2. Schizophrenia  3. Anxiety disorder  4. Depression | 20126, 20122, 20124, 20125, 20123, 1980, 2010 |
| Medications | UK Biobank touchscreen questionnaire about health and medical history at baseline, and the inclusion relationships of some drugs are as follows:  1. NASIDs: acetaminophen, ibuprofen, or aspirin  2. Acid inhibitors: PPIs or H2 receptor agonist  3. Hypolipidemic drugs: statins or cholesterol-lowering drugs. | 10004, 6155, 10007, 6179, 10723, 6177, 6153, 6154 |

Abbreviation: NSAIDs: nonsteroidal anti-inflammatory drugs. PPIs: proton pump inhibitors. UKB: UK Biobank.

**Supplementary Table S3.** Sensitivity analysis regarding risk of IBS according to cholecystectomy.

| **Sensitivity analysis** | **No. of IBS** | **No. of participants** | **Adjusted HR (95%CI)** | ***p*-value** |
| --- | --- | --- | --- | --- |
| **1.** Propensity score matching cohort between cholecystectomy and non-cholecystectomy (**1:4 matching, N=29811**). | | | | |
| Non-cholecystectomy | 1080 | 22928 | 1.00 (Reference) |  |
| Cholecystectomy | 485 | 6883 | 1.42 (1.28-1.59) | <0.001 |
| **2.** Excluding IBS participants diagnosed within 2 years after baseline (**N=411656**). | | | | |
| Non-cholecystectomy | 14248 | 398556 | 1.00 (Reference) |  |
| Cholecystectomy | 858 | 13100 | 1.45 (1.32-1.60) | <0.001 |
| **3**. Excluding IBS participants diagnosed within 4 years after baseline (**N=408648**). | | | | |
| Non-cholecystectomy | 13736 | 395678 | 1.00 (Reference) |  |
| Cholecystectomy | 810 | 12970 | 1.43 (1.30-1.59) | <0.001 |

Note: All adjusted HRs were adjusted for the following covariates: age (continuous variable), gender (female or male), race (White or non-White), education level (college/university or non-college/university), Townsend deprivation index (continuous variable), physical activity (continuous variable), smoking (yes or no), alcohol drinking (yes or no), healthy diet (yes or no), quality sleep (yes or no), BMI (continuous variable), type 2 diabetes (yes or no), hypertension (yes or no), hyperlipidemia (yes or no), mental illness (yes or no), gallstones (yes or no), NASID intake (yes or no), acid inhibitor intake (yes or no), hypoglycemic drug intake (yes or no), hypolipidemic drug intake (yes or no), antihypertensive drug intake (yes or no), vitamin supplement intake (yes or no), and mineral supplement intake (yes or no). Abbreviations: IBS, irritable bowel syndrome. HR, hazard ratio. CI, confidence interval.

| **Characteristic** | **Total** | **Cholecystectomy** | | ***p-*value** |
| --- | --- | --- | --- | --- |
|  | **(N=29811)** | **No**  **(N=22928)** | **Yes**  **(N=6883)** |  |
| **Mean (SD) age (years)** | 59.91 (7.00) | 59.94 (6.97) | 59.80 (7.09) | 0.141* |
| **Male, No. (%)** | 6713 (22.5) | 5149 (22.5) | 1564 (22.7) | 0.656 |
| **White race, No. (%)** | 28826 (96.7) | 22167 (96.7) | 6659 (96.7) | 0.822 |
| **College/University, No. (%)** | 7634 (25.6) | 5887 (25.7) | 1747 (25.4) | 0.634 |
| **Media (IQR) Townsend Deprivation Index** | 12.93 (15.36) | 12.91 (15.22) | 13.00 (15.64) | 0.181^※^ |
| **Smoking, No. (%)** | 14156 (47.5) | 10903 (47.6) | 3253 (47.3) | 0.681 |
| **Alcohol Drinking, No. (%)** | 21467 (72.0) | 16644 (72.6) | 4823 (70.1) | <0.001 |
| **Healthy diet, No. (%)** | 20221 (67.8) | 15619 (68.1) | 4602 (66.9) | 0.051 |
| **Quality sleep, No. (%)** | 10883 (36.5) | 8401 (36.6) | 2482 (36.1) | 0.388 |
| **Media (IQR) Physical activity**  **(MET hours/week)** | 40.95 (28.48) | 40.95 (28.38) | 41.10 (28.78) | 0.270^※^ |
| **Mean (SD) BMI** | 29.59 (5.93) | 29.50 (5.93) | 29.88 (5.89) | <0.001* |
| **Type 2 diabetes, No. (%)** | 7542 (25.3) | 5727 (25.0) | 1815 (26.4) | 0.021 |
| **Hypertension, No. (%)** | 23368 (78.4) | 17962 (78.3) | 5406 (78.5) | 0.736 |
| **Hyperlipidemia, No. (%)** | 16778 (56.3) | 12868 (56.1) | 3910 (56.8) | 0.323 |
| **Mental illness, No. (%)** | 2151 (7.2) | 1644 (7.2) | 507 (7.4) | 0.600 |
| **Gallstones, No. (%)** | 3069 (10.3) | 1534 (6.7) | 1535 (22.3) | <0.001 |
| **NASID intake, No. (%)** | 14677 (49.2) | 11251 (49.1) | 3426 (49.8) | 0.312 |
| **Acid inhibitor intake, No. (%)** | 3902 (13.1) | 2874 (12.5) | 1028 (14.9) | <0.001 |
| **Hypoglycemic drug intake, No. (%)** | 780 (2.6) | 576 (2.5) | 204 (3.0) | 0.044 |
| **Hypolipidemic drug intake, No. (%)** | 6995 (23.5) | 5330 (23.2) | 1665 (24.2) | 0.109 |
| **Antihypertensive drug intake, No. (%)** | 8794 (29.5) | 6704 (29.2) | 2090 (30.4) | 0.075 |
| **Vitamin supplement intake, No. (%)** | 4750 (15.9) | 3683 (16.1) | 1067 (15.5) | 0.273 |
| **Mineral supplement intake, No. (%)** | 6863 (23.0) | 5303 (23.1) | 1560 (22.7) | 0.432 |

**Supplementary Table S4**. Baseline characteristics according to cholecystectomy after 1:4 propensity score matching.

Note: Normally distributed continuous variables displayed as the means (SDs), nonnormally distributed continuous variables displayed as medians (IQRs), and categorical variables displayed as numbers (percentages). *: P value calculated using independent samples Student’s t-test. ※: P value calculated using Mann-Whitney U test. The remaining P value calculated using Pearson χ^2^ test. Abbreviations: SD, standard deviation. IQR, interquartile range. MET, metabolic equivalent. BMI, body mass index. NASID, nonsteroidal anti-inflammatory drug.

**Reference**

1. Altman DG, Andersen PK. Calculating the number needed to treat for trials where the outcome is time to an event. Bmj 1999;319:1492-5.

2. Lourida I, Hannon E, Littlejohns TJ, et al. Association of Lifestyle and Genetic Risk With Incidence of Dementia. JAMA 2019;322:430-437.

3. Nishiyama Y, Fujikawa T. Tea Consumption and All-Cause and Cause-Specific Mortality in the UK Biobank. Ann Intern Med 2023;176:eL220477.

4. Liu D, Li ZH, Shen D, et al. Association of Sugar-Sweetened, Artificially Sweetened, and Unsweetened Coffee Consumption With All-Cause and Cause-Specific Mortality : A Large Prospective Cohort Study. Ann Intern Med 2022;175:909-917.

5. Watson NF, Badr MS, Belenky G, et al. Recommended Amount of Sleep for a Healthy Adult: A Joint Consensus Statement of the American Academy of Sleep Medicine and Sleep Research Society. J Clin Sleep Med 2015;11:591-2.
